# High Satisfaction and Strength Recovery After Mini-Open Double-Row Repair of Partial Gluteal Tears Without Advanced Osteoarthritis: A Unicentric Retrospective Cohort Study

**DOI:** 10.3390/medicina61101863

**Published:** 2025-10-16

**Authors:** Ingo J. Banke, Amr Seyam, Kilian Blobner, Rüdiger von Eisenhart-Rothe, Vanessa Twardy

**Affiliations:** Clinic of Orthopaedics and Sports Orthopaedics, School of Medicine and Health, TUM University Hospital, Technical University of Munich, Ismaninger Str. 22, 81675 Munich, Germany; seyamamr@gmail.com (A.S.); kilian.blobner@mri.tum.de (K.B.); eisenhart@tum.de (R.v.E.-R.); vanessa.twardy@mri.tum.de (V.T.)

**Keywords:** gluteal tendon tear, hip abductor repair, double-row technique, hip bridge, patient satisfaction, isometric strength, non-arthritic hip

## Abstract

*Background and Objectives*: Partial gluteal tendon tears in native hips are often misdiagnosed as greater trochanteric pain syndrome, resulting in ineffective conservative treatment and persistent symptoms. Although surgical repair techniques exist, data on objective strength outcomes in non-arthritic hips remain limited. The objective of this study was to evaluate pain reduction, patient-reported outcomes (PROMs), and isometric hip abductor strength following mini-open, knotless double-row repair using the Hip Bridge technique. *Material and Methods*: This retrospective, single-center cohort study (Level III) with prospective outcome evaluation included 27 patients (mean age 53 years, BMI 27 kg/m^2^) with partial gluteal tendon tears and no advanced osteoarthritis (Tönnis grade ≤ 1), treated between 2015 and 2022 using the mini-open, knotless double-row Hip Bridge technique. The mean follow-up was 29.3 ± 24.3 months (minimum 6 months). Diagnosis was confirmed by 3-Tesla MRI, and other sources of lateral hip pain were excluded. Clinical outcomes included the Visual Analog Scale (VAS), modified Harris Hip Score (mHHS), Hip Outcome Score (HOS), normalized Western Ontario and McMaster Universities Osteoarthritis Index (nWOMAC), and Copenhagen Hip and Groin Outcome Score (HAGOS). Isometric hip abductor strength was assessed in 22 patients using a dynamometer, comparing the operated and contralateral limbs. *Results*: Postoperative satisfaction was high: 93% would undergo surgery again, 88% reported improved Trendelenburg gait, and 85% noted subjective strength gains. Pain improved significantly from VAS 8 (range, 3 to 10) preoperatively to VAS 2 (range, 0 to 7) postoperatively (*p* < 0.001); 100% reported pain relief. Patient-reported outcome scores were mHHS, 84.2; nWOMAC, 86.5; HOS, 80.7; and HAGOS, 70.7. Isometric strength testing showed significant improvement on the operated side (Fmax: *p* = 0.006; Fmean: *p* = 0.009). The mean limb symmetry index was 118% for Fmax and 122% for Fmean. *Conclusions*: Mini-open, knotless double-row repair of partial gluteal tears in non-arthritic hips yields adequate pain relief, high satisfaction, and objective strength recovery. The Hip Bridge technique could be an effective option after failed conservative treatment. Future prospective comparative studies are warranted to validate mid-term outcomes and establish long-term efficacy.

## 1. Introduction

Gluteal tendon tears—especially those affecting the gluteus medius (GMed) and minimus (GMin)—are increasingly recognized as a distinct clinical entity. However, partial gluteal tears in native hips remain frequently misdiagnosed as greater trochanteric pain syndrome (GTPS), a term that encompasses a broad spectrum of lateral hip pathologies [1]. As a result, many patients receive prolonged but ineffective conservative treatments, leading to persistent pain, reduced quality of life, and delayed surgical care [2].

Recent imaging and anatomical studies have shown that partial gluteal tears may affect up to 10% of adults over 60 years old, often due to degenerative changes rather than acute trauma [3]. Analogous to rotator cuff pathology in the shoulder, gluteal tendon injuries have been described as the “rotator cuff tears of the hip” [4], with similar biomechanical implications and surgical strategies [5]. Overlooked gluteal tendon tears may lead to progressive fatty degeneration and reduced functional outcome [2]. Clinically, patients may present with a limping gait (Trendelenburg gait) and/or significant weakening of the hip abductor musculature [3].

Several surgical techniques—open or endoscopic, single-row or double-row—have been described for repairing gluteal tendon tears [6,7,8]. Double-row repair, widely used in rotator cuff surgery, has demonstrated superior tendon-to-bone contact area and biomechanical stability [5]. The mini-open, knotless double-row Hip Bridge technique, as described by Gollwitzer et al. [9], aims to reproduce these principles at the hip using a less invasive approach. However, the current literature on outcomes following gluteal repair remains heterogeneous in terms of patient population (with or without total hip arthroplasty), surgical technique, and reporting of objective strength data [2].

Objective strength measures are rarely reported, likely because reliable assessment requires standardized testing protocols and validated dynamometers, which are not routinely available in clinical practice. To date, few studies have quantified postoperative hip abductor strength or examined outcomes specifically in native hips without advanced osteoarthritis.

This study aimed to evaluate objective recovery of hip abductor strength with a validated dynamometer, pain reduction, and patient-reported outcome measures (PROMs), following mini-open, knotless double-row repair using the Hip Bridge technique. We hypothesized that this technique would result in high satisfaction, significant pain reduction, and restoration of hip abductor strength to at least match the contralateral limb. We further acknowledged that limb symmetry indices greater than 100% could occur due to contralateral subclinical degeneration.

## 2. Materials and Methods

### 2.1. Study Design

The study was designed as a retrospective, unicentric cohort study (Level III) with prospective outcome evaluation conducted at our institution. Ethical approval was obtained from the local ethics committee, and written informed consent was provided by all patients prior to inclusion.

All consecutive patients treated with mini-open double-row repair of partial gluteal tendon tears in native hips between October 2015 and September 2022 were considered for inclusion. All procedures were performed by a single senior surgeon. Inclusion criteria were patients with partial gluteal tendon tears confirmed by 3-Tesla MRI and without advanced osteoarthritis (Tönnis grade ≤ 1). Exclusion criteria were patients who declined participation, postoperative infection, prior ipsilateral hip arthroplasty, advanced osteoarthritis (Tönnis grade ≥ 2), or any additional ipsilateral hip surgery following the repair.

The sample size was not predetermined. Instead, all consecutive patients who fulfilled the inclusion criteria during the study period were included. This consecutive sampling approach ensured that the cohort was representative of all eligible cases at our center.

### 2.2. Imaging

**Figure 1:**
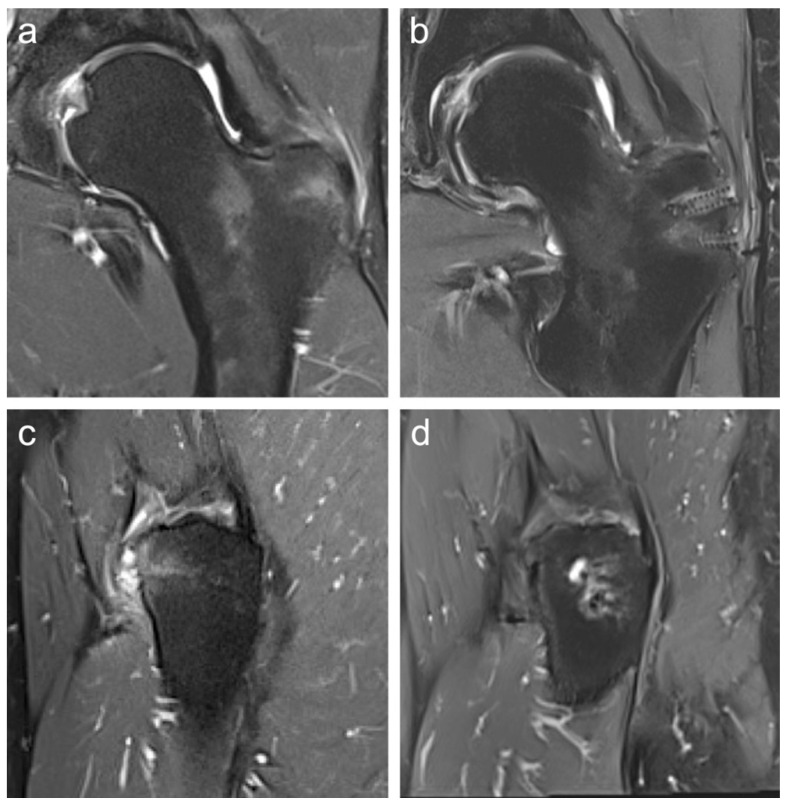
Preoperative coronal (**a**) and sagittal (**c**) 3-Tesla MRI showing a partial rupture of the GMin and GMed tendon insertions at the greater trochanter. Postoperative coronal (**b**) and sagittal (**d**) 3-Tesla MRI confirm intact suture anchor placement, complete tendon reattachment, and absence of re-rupture, seroma, or recurrent bursitis.

Preoperative imaging included 3-Tesla magnetic resonance imaging (MRI) (Figure 1a,c) and standard radiographs in all patients to confirm the diagnosis, assess muscle quality, and exclude other pathologies such as isolated trochanteric bursitis without involvement of the gluteal tendon footprint. Postoperative MRI was performed to confirm the integrity of the suture anchors, verify tendon reattachment at the gluteal footprint, and rule out re-rupture, seroma, or recurrent bursitis (Figure 1b,d).

### 2.3. Surgical Technique

**Figure 2:**
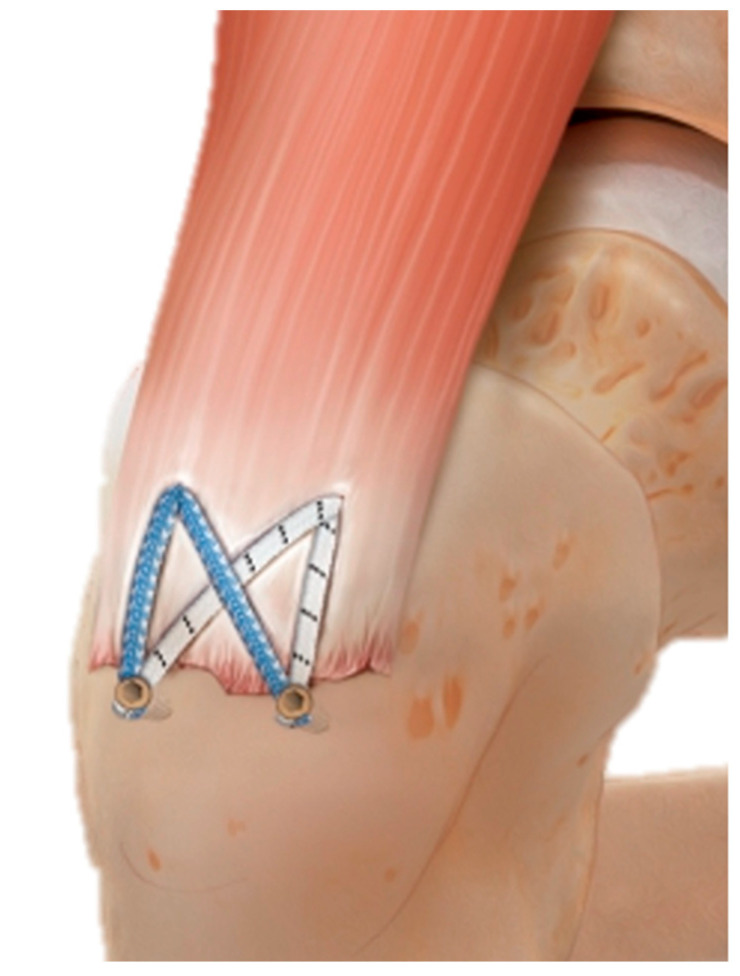
The figure graphically demonstrates the anchor and suture configuration of the knotless double-row “Hip Bridge” technique described by Twardy et al. [11].

The patient was placed in the lateral decubitus position. A lateral skin incision (approximately 6 cm) was made, starting 2 cm proximal to the tip of the greater trochanter and aligned distally along the femoral axis. After subcutaneous dissection, the iliotibial band was split longitudinally, and a bursectomy was performed. The gluteal tendon tear was identified, mobilized, and the anatomical footprint debrided. Two PEEK suture anchors (SwiveLock^®^, 4.75 mm, Arthrex, Naples, FL, USA), each preloaded with FiberTape^®^ (2 mm, Arthrex, Naples, FL, USA), were inserted proximally at 90° into the gluteal tendon footprint. The FiberTapes^®^ were passed through the tendon and then distally secured: one limb of each proximal FiberTape^®^ was routed into two additional SwiveLock^®^ anchors placed approximately 20 mm distal to the first row (Figure 2). Tension was applied to compress the tendon against the bone before final fixation. This Hip Bridge construct is a modification of the minimally invasive SpeedBridge™ technique originally developed for rotator cuff repair in the shoulder [10].

### 2.4. Postoperative Care

Patients were discharged two days after surgery. For the first two weeks, partial weight-bearing of 20 kg on crutches was prescribed. Thereafter, weight-bearing was increased as tolerated based on pain. To protect the repair, patients wore an abduction-limiting brace for six weeks, restricting adduction beyond neutral and prohibiting active abduction. High-impact activities were not permitted for three months postoperatively.

To reduce the risk of heterotopic ossification, all patients received anti-inflammatory prophylaxis with Celecoxib 100 mg twice daily for 21 days.

### 2.5. Postoperative Assessment

Pain levels were routinely documented preoperatively and assessed at postoperative follow-up (mean 29.3 ± 24.3 months; range 6–85 months) using the Visual Analog Scale (VAS) for pain (0–10 scale). Patient-reported outcome measures (PROMs), including the modified Harris Hip Score (mHHS), Copenhagen Hip and Groin Outcome Score (HAGOS), Hip Outcome Score (HOS-ADL), and the normalized Western Ontario and McMaster Universities Osteoarthritis Index (nWOMAC), were collected postoperatively only (mean 29.3 ± 24.3 months; range 6–85 months) to quantify subjective functional outcome [12]. Higher nWOMAC scores (max. 100) indicate fewer symptoms, including reduced pain, stiffness, and functional limitations.

### 2.6. Objective Hip Abduction Strength Evaluation

**Figure 3:**
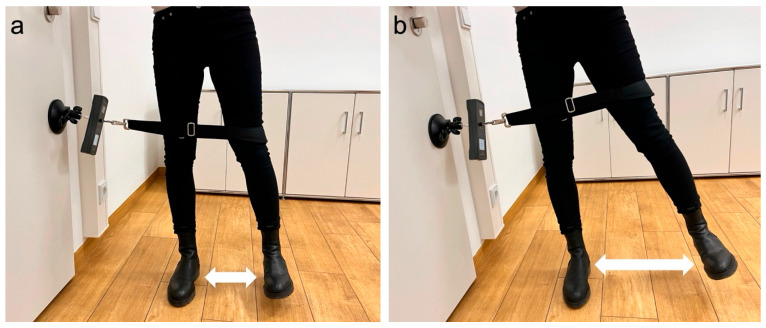
Measurement of isometric hip abduction strength: (**a**) Patient positioned standing upright with extended legs. A measuring strap was positioned at the level of the lateral femoral condyle, 10 cm proximal to the joint line. (**b**) Each leg was tested three times with five-second maximal isometric contractions.

To assess general hip mobility, objective measurements—including passive range of motion (ROM)—were taken prior to each strength test. All patients received a brief standardized instruction, but performed the test without a warm-up to reflect everyday functional capacity. Objective hip abductor strength was assessed in a standardized protocol using the IsoForceControl^®^ EVO2 dynamometer (IsoForceControl^®^ EVO2, Herkules Kunststoff AG, Oberburg, Germany). The IsoForceControl^®^ dynamometer has previously demonstrated high test–retest reliability and validity for isometric strength testing, supporting its use in this context [13,14]. Patients were positioned standing upright with extended legs and performed three maximal isometric hip abduction contractions per side, each held for five seconds (Figure 3). In accordance with Ebert et al. [2], all measurements were performed in a standing position with the leg extended, as this setup more accurately reflects everyday functional conditions. The maximum (Fmax) and mean (Fmean) force were recorded, and side-to-side differences were expressed as a limb symmetry index (LSI) to account for interindividual variability. In addition, strength was clinically graded using the Medical Research Council (MRC) scale (0–5).

### 2.7. Statistical Analysis

All analyses were performed in SPSS v29.0 (IBM, New York, NY, USA). Two-tailed tests with α = 0.05 were used throughout. Isometric hip abductor strength (Fmax, Fmean): Operated vs. contralateral limbs were compared within subjects. Normality of paired differences was assessed by Shapiro–Wilk and visual inspection of Q-Q plots. As this assumption was reasonably met, we used paired *t*-tests, and the results are reported as means ± SD. Pain (VAS/NRS 0–10): Within-subject pre- vs. postoperative comparisons were analyzed using the Wilcoxon signed-rank test. Pain scores did not meet normality assumptions (Shapiro–Wilk and Q-Q plot inspection); therefore, nonparametric inference was applied. Results are reported as median with range and exact two-tailed *p*-values. Exploratory subgroup comparisons (e.g., follow-up > 12 vs. ≤12 months) were analyzed primarily with the Mann–Whitney U test owing to small samples and potential non-normality; unpaired *t*-tests were used as sensitivity analyses when distributional assumptions were reasonably satisfied, yielding concordant inferences. As the study was not designed or powered to identify independent risk factors, all subgroup analyses were exploratory and unadjusted and were conducted using the between-group tests described above. PROMs (mHHS, nWOMAC 0–100 [100 = best], HOS-ADL, HAGOS) were collected postoperatively only and presented descriptively (means ± SD for comparability with prior literature); no inferential tests were performed for PROMs. No adjustments for multiplicity were applied, given the limited number of primary outcomes. A post hoc power check for the paired strength outcomes (based on the observed paired differences) indicated power = 0.83 (β = 0.17) for Fmax and 0.78 (β = 0.22) for Fmean at α = 0.05; these calculations are ancillary and do not alter inference, which is based on the reported *p*-values.

## 3. Results

**Figure 4:**
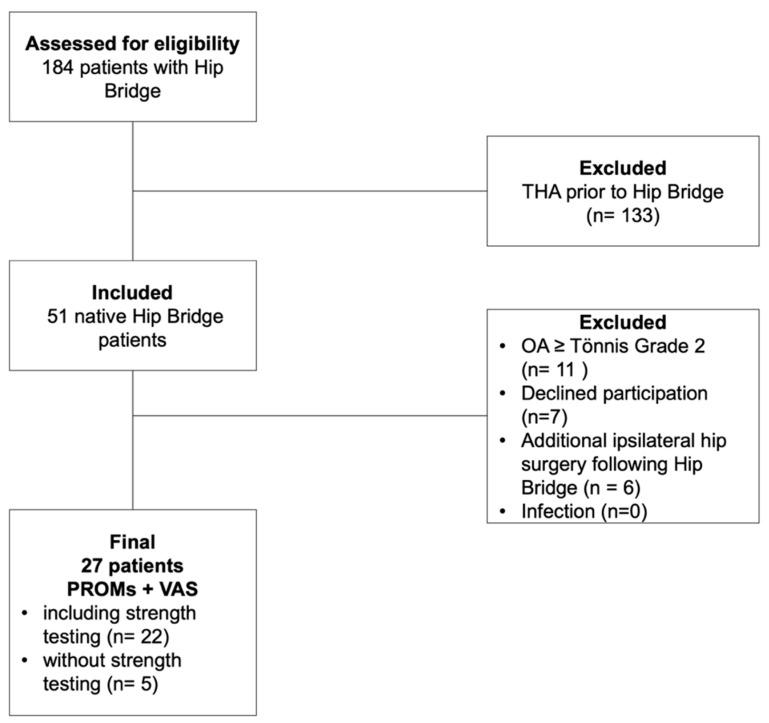
Flow diagram of patient inclusion and exclusion. The diagram shows the number of patients assessed for eligibility, the reasons for exclusion, the number of patients included, and those analyzed in the final evaluation.

Taking into account the exclusion criteria, 27 patients were eligible for inclusion in the study. Of these, five patients were unable to participate in the isometric strength testing due to relocation. These patients were excluded from the strength analysis but included in the evaluation of PROMs, which they completed via telephone interview and postal questionnaire (Figure 4).

The mean follow-up was 29.3 ± 24.3 months, with a minimum of 6 months (range, 6–85 months). Preoperatively, all patients presented with characteristic lateral hip pain during active abduction or when lying on the affected side. All had undergone a structured course of conservative treatment for at least 6 months, including professional physiotherapy and guided self-exercises based on a standardized training program. Patient demographics are summarized in Table 1. Demographic data were collected preoperatively.

All patients underwent preoperative 3-Tesla magnetic resonance imaging (MRI) to assess the gluteal tendons. Degenerative tendon tears were identified in 85.2% of cases, while 14.8% were associated with an (additional) traumatic event. According to the Goutallier–Fuchs classification of fatty infiltration, 92.6% of patients were graded 0–1, and 7.4% were classified as possibly Grade 2, though assessment was limited due to suboptimal image quality. Partial ruptures were present in 88% of cases on preoperative imaging, while the remaining 12% showed more isolated tendinopathy without a clear tear. However, intraoperative findings confirmed partial tendon rupture in all patients. The GMin was affected in 30% of patients in isolation, while combined GMin and GMed lesions were found in 62%. Bilateral hip MRI was available in 16 patients. On the contralateral side, 50% (8 patients) showed signs of gluteal insufficiency of lesser severity compared to the symptomatic side. In 7 patients (43%), MRI findings were symmetrical. One patient (6%) exhibited a more advanced, yet asymptomatic gluteal lesion on the contralateral side (Table 2).

Findings from preoperative 3-Tesla MRI of the operated hip and, where available, the contralateral hip (n = 16). Results are given as percentages. Fatty infiltration was graded according to the Goutallier–Fuchs classification. Intraoperative assessment confirmed a partial tendon rupture in all patients.

Pain improved significantly from a median VAS 8 (range, 3 to 10) preoperatively to VAS 2 (range, 0 to 7) at final follow-up (*p* < 0.001) (Table 3). All patients (100%) reported at least some pain relief. The overall patient satisfaction was high, with 93% of patients stating they would undergo the surgery again. Postoperative Patient-reported outcome scores at follow-up were mean mHHS 84.2 ± 18.5, nWOMAC 86.5 ± 15.4, HOS-ADL 80.7 ± 17.5, and HAGOS 70.7 ± 22.2 (Table 3). No preoperative PROM scores were available for direct comparison due to the retrospective design. During clinical examination, all patients demonstrated good hip function (hip flexion ≥ 110° and internal rotation ≥ 20° on the operated side).

Isometric abductor strength was assessed bilaterally only in 22 patients due to relocation. The operated limb showed a significant restoration of strength compared to the contralateral side. Fmax was 138.8 ± 55.8 N on the operated side versus 121.7 ± 55.6 N on the contralateral side (*p* < 0.006), while Fmean reached 116.2 ± 50.3 N versus 100.1 ± 48.7 N (*p* < 0.009) (Table 4). The range of measured strength varied widely, from 41.6 N to 259.3 N on the operated side and from 34.1 N to 284.1 N on the contralateral side (Table 4).

This corresponded to a mean limb symmetry index (LSI) of 118% for Fmax and 122% for Fmean, indicating that, on average, the operated limb achieved equal or greater strength than the unaffected limb. However, it should be noted that all patients who underwent bilateral MRI revealed some form of contralateral gluteal tendon degeneration. This may have influenced the comparison and contributed to the observed side-to-side differences. Due to the known high variability in individual strength levels, comparisons were performed within subjects rather than between patients, as previously recommended [15]. To confirm the observed differences, statistical analysis was conducted on the individual strength differences (contralateral minus operated limb), which also showed statistical significance (*p* < 0.006 for Fmax, *p* < 0.009 for Fmean) (Table 4). According to the Medical Research Council (MRC) scale, all patients demonstrated normal hip abduction strength (5/5) on both limbs at final follow-up. To contextualize the strength measurements, isometric hip abductor strength was also assessed in 20 healthy control subjects bilaterally. In this group, both maximum and average force showed minor side-to-side differences. Absolute strength values were moderately higher than in the patient cohort, but within a comparable range—most likely reflecting the younger age and generally higher activity level of the healthy control group.

No significant association was found between postoperative pain levels and the patient’s willingness to undergo the surgery again (*p* = 0.752). Additionally, 88% of patients reported an improvement in Trendelenburg gait, and 85% reported a subjective increase in hip abductor strength. There was no significant association between objectively measured strength and the decision to undergo surgery again (*p* = 0.312 for Fmax, *p* = 0.216 for Fmean).

Exploratory subgroup comparisons were performed to assess possible unadjusted associations between baseline characteristics and postoperative outcomes. There was no significant difference in postoperative hip abductor strength (*p* = 0.873) or pain levels (*p* = 0.828) between patients with follow-up durations longer or shorter than 12 months. A total of 4 out of 27 patients (14.8%) had undergone prior surgery on the ipsilateral hip—specifically, iliotibial tract lengthening via Z-plasty combined with bursectomy—before gluteal tendon repair (Table 1). However, no significant association was found between prior surgery and postoperative strength outcomes (*p* = 0.110). There was also no statistically significant association between hip abductor strength (Fmax) and potential influencing factors such as age (*p* = 0.292), BMI (*p* = 0.990), duration since surgery (*p* = 0.668), smoking status (*p* = 0.256), affected side (*p* = 0.391), or postoperative pain (*p* = 0.763). No statistically significant associations were found for any of the examined variables. All employed patients successfully returned to work. Return to sports was not systematically assessed due to the relatively older and less athletic patient population. At the time of final follow-up, no revision surgeries had been performed. Furthermore, no major complications were observed, including re-ruptures, infections, or neurovascular injury.

## 4. Discussion

Our previous biomechanical investigations have already demonstrated the superior primary stability of the Hip Bridge construct when compared with the Mason–Allen suture technique [11]. The findings of this study confirm that mini-open, knotless double-row repair using the Hip Bridge technique is a safe and effective surgical option for partial gluteal tendon tears in non-arthritic hips. All patients reported pain reduction, and 93% would choose to undergo the procedure again.

Unlike most existing studies, our cohort consisted of younger patients (mean age 52 years) with native hips and no advanced osteoarthritis. Most patients had degenerative tears and considerable functional limitations due to Trendelenburg gait and hip abductor weakness—particularly relevant in physically active individuals. In this context, restoration of hip strength is a critical outcome.

To quantify this, we used objective dynamometric testing in a standing position as described by Ebert et al. [16], which better reflects everyday function than supine testing. By applying a validated dynamometric protocol in our study, we aimed to provide objective data on hip abductor strength recovery. Our mean LSI was 118% (Fmax) and 122% (Fmean), suggesting slightly higher strength in the operated limb. This finding should be interpreted with caution, as some form of contralateral gluteal tendon degeneration was present in all patients who underwent bilateral MRI. Such degeneration may reduce the apparent strength of the non-operated side and thus bias the limb-to-limb comparison. Ebert et al. [16] similarly reported a mean LSI of 128.5%. Bagger Bohn et al. further support the use of objective strength testing [17]. In their cohort, 69% of patients achieved an LSI ≥ 95% at 12-month follow-up. They also demonstrated that persistent Trendelenburg gait correlated with lower strength outcomes, which matches our finding that 88% of patients improved in this respect. Capurro-Soler et al. investigated open double-row repair with tensor fascia lata augmentation in a small cohort (n = 8), reporting marked functional improvements (HHS from 48 to 94; iHOT from 20 to 83) and preserved strength over 12 months—despite differences in technique, their results align well with ours [18]. Similarly, Harper et al. reported significant strength improvement and better healing in a comparison of tenodesis versus bone–trough repair, confirming the broader trend of strength recovery after gluteal repair [19]. Navas et al. reported a 100% return to recreational activity following open gluteal reconstruction [20]. Though strength was assessed subjectively, their findings support the general trend of high functional restoration after open repair. The importance of early surgical intervention for partial ruptures is also supported by Ebert et al., who found normal strength recovery in 73% of partial tears compared to 46% in complete ruptures—matching our findings in a comparable subgroup [16]. In contrast to studies that included patients with total hip arthroplasty (THA), our cohort was limited to native hips. Makridis et al. reported similar outcomes, but their cohort was older and more heterogeneous [21]. Barrera et al. also studied mini-open repair but did not include objective strength measurements [6]. The 2015 review by Ebert et al. highlighted the lack of strength data in this area, which our study helps to address [2]. Finally, to contextualize the findings, we also measured hip abductor strength in healthy controls. Although the absolute values were moderately higher, the side-to-side difference was minimal, confirming the clinical relevance of our strength recovery results.

### Limitations

This study has several limitations. The retrospective design and modest sample size introduce risk of bias and limit generalizability, though this was partially mitigated by a single-center, single-surgeon setting and homogeneous inclusion criteria (native hips without advanced osteoarthritis). The condition studied—native gluteal insufficiency without osteoarthritis—is relatively uncommon yet highly symptomatic, which also contributed to the limited cohort size. Preoperative PROMs were not available, restricting longitudinal comparison of patient-reported outcomes. The cohort was predominantly female, reflecting the epidemiology of gluteal tendon tears but limiting sex-based comparisons. Follow-up duration was heterogeneous (6–85 months), and return to sports or specific functional activities was not systematically assessed. A mini-open approach was used rather than an endoscopic technique; while the literature suggests comparable outcomes, direct technique comparisons were beyond the scope of this study. The subgroup analyses were exploratory and limited to unadjusted comparisons; therefore, the observed relationships should not be interpreted as independent risk factors. Although all patients had at least 6 months of follow-up, longer-term tendon durability remains to be confirmed. Nevertheless, no early re-ruptures or major complications were observed, making delayed failure less likely.

Future studies are needed. In particular, prospective comparative trials should evaluate mini-open versus endoscopic repair, as well as single-row versus double-row techniques. Long-term follow-up is warranted to determine the durability of tendon healing and sustained functional outcomes.

## 5. Conclusions

Mini-open Hip Bridge repair of partial gluteal tendon tears resulted in substantial pain relief, high patient satisfaction, and restoration of hip abductor strength. These findings support consideration of this procedure as a possible treatment option for non-arthritic patients after failed conservative therapy. Prospective studies with long-term follow-up are required to validate and extend our observations.

## Figures and Tables

**Table 1:** Baseline characteristics of patients undergoing mini-open knotless double-row hip bridge repair for partial gluteal tendon tears.

	Number of Patients
Number of patients (N)	27
Sex (male vs. female)	5/22
Age at surgery (years)	52.6 ± 16.1
BMI (kg/m^2^)	27.0 ± 4.7
Smoking Prior surgery on the affected side *	22% 14.8%
Affected side (right vs. left side) Contralateral total hip arthroplasty	14/13 1
Tönnis Grad 0 Tönnis Grad 1	60% 40%

Continuous variables are presented as mean ± SD, categorical variables as n (%). Demographic data were collected preoperatively. * Prior surgery on the affected side refers to iliotibial tract Z-plasty with bursectomy. Tönnis grades refer to the operated hip.

**Table 2:** Preoperative 3-Tesla MRI findings of the operated and contralateral hips in the study cohort.

Type of Lesion	Proportion
Degenerative tendon lesion	85.2%
Traumatic (additional)	14.8%
**Goutallier–Fuchs Classification**	
Fatty infiltration Grade 0–1	92.6%
Fatty infiltration Grade 2	7.4%
**Type of Rupture**	
Partial rupture	88%
GMin GMin + GMed Tendinopathy without clear rupture	30% 62% 12%
**Contralateral side (n = 16)**	
Contralateral insufficiency (less severe)	50%
Symmetrical findings	44%
Advanced asymptomatic contralateral lesion	6%

**Table 3:** Postoperative clinical outcomes: pain, satisfaction, and PROMs (mHHS, nWOMAC, HOS-ADL, and HAGOS).

	Results
VAS preoperative	VAS 8 (range, 3 to 10) *
VAS postoperative	VAS 2 (range, 0 to 7) *
Reduction in Pain	100%
Patients would do surgery again	93%
Improvement in Trendelenburg gait	88%
Improvement of hip abductor strength	85%
(subjective)	
mHHS nWOMAC HOS (ADL) HAGOS	82.2 ± 16.0 86.5 ± 15.4 80.7 ± 17.5 70.7 ± 22.2
Rate of major complications	0%

Clinical outcomes (mean 29.3 ± 24.3 months; minimum 6 months). VAS pain is reported pre- and postoperatively; PROMs (mHHS, nWOMAC, HOS-ADL, and HAGOS) were assessed postoperatively only. Higher nWOMAC scores indicate fewer symptoms. Data are reported as medians with ranges and/or means ± SD or n (%), as appropriate. Comparative testing was performed as described in Statistical Methods; * indicates a significant VAS pre- vs. postoperative difference (*p* < 0.001).

**Table 4:** Postoperative isometric hip abductor strength: operated versus contralateral limb (paired within-subject comparison, N = 22).

Group	Gluteal Repair (N)	Contralateral Limb (N)	*t*-Test *p*-Value
Mean isometric strength force	116.2 ± 50.3	100.1 ± 48.7	*p* < 0.009 *
Maximum isometric strength force	138.8 ± 55.8	121.7 ± 55.6	*p* < 0.006 *
Individual strength difference (Fmean)	−16.0 ± 26.3		*p* < 0.009 *
Individual strength difference (Fmax)	−17.1 ± 26.0		*p* < 0.006 *
Minimum	41.6	34.1	
Maximum	259.3	284.1	

Isometric hip abductor strength (N) measured in the standing position with a validated dynamometer; three 5 s maximal isometric contractions per limb. Values are mean ± SD, with minimum and maximum values and individual side-to-side differences. Comparisons are between operated and contralateral limbs, as detailed in Statistical Methods; * denotes statistically significant difference.

## Data Availability

The underlying data of this study are available from the corresponding author upon reasonable request.

## Abbreviations

Fmax	Maximum force
Fmean	Average force
GMin	Musculus gluteus minimus
GMed	Musculus gluteus medius
GMax	Musculus gluteus maximus
HAGOS	Copenhagen Hip and Groin Outcome Score
HOS	Hip Outcome Score
LSI	Limb Symmetry Index
mHHS	modified Harris Hip Score
MRC	Medical Research Council
MRI	Magnetic Resonance Imaging
OA	Osteoarthrosis
PEEK	Polyetheretherketone
THA	Total Hip Arthroplasty
VAS	Visual Analog Scale
nWOMAC	normalized Western Ontario and McMaster Universities Osteoarthritis Index
